# Behçet’s disease: incidence, prevalence, and real-word data on the use of biologic agents in Japan

**DOI:** 10.1007/s00535-024-02191-y

**Published:** 2024-12-06

**Authors:** Tadakazu Hisamatsu, Makoto Naganuma, Philippe Pinton, Mitsuhiro Takeno

**Affiliations:** 1 Department of Gastroenterology and Hepatology, Kyorin University School of Medicine, Tokyo, Japan; 2 Division of Gastroenterology and Hepatology, Third Department of Internal Medicine, Kansai Medical University, Hirakata, Osaka, Japan; 3 Clinical and Translational Sciences, Ferring Pharmaceuticals, 2770 Kastrup, Denmark; 4 Department of Allergy and Rheumatology, Nippon Medical School Musashi Kosugi Hospital, Kawasaki, Kanagawa, Japan

**Keywords:** Behçet’s disease, Gastrointestinal Behçet’s disease, Treatment trends, TNFαi, Japan

## Abstract

**Background:**

Behçet’s disease (BD) is an autoinflammatory disease that can affect multiple organs, including the gastrointestinal tract. Conventional management comprises anti-inflammatory drugs such as glucocorticoids (GCs) and/or immunomodulators that alleviate symptoms. The introduction of biological agents that target tumor necrosis factor α (TNF-α) has improved disease management. The goal of this work was to analyze the current prevalence and incidence of total BD and gastrointestinal Behçet’s disease (GIBD) in Japan, and examine treatment trends, especially regarding the use of TNF-α inhibitors (TNFαi).

**Methods:**

We performed a retrospective descriptive observational study in which BD and GIBD demographic trends, medical treatment patterns, and reported adverse events (AEs) were assessed among patients with data recorded between 2017 and 2021 in the Japan Medical Data Center Claims Database (now JMDC Inc.).

**Results:**

Prevalence of BD and GIBD in Japan during the observation period increased at an annual rate of + 3% and + 4%, respectively, while incidence decreased by − 5% and − 2%, with a more prominent decline in confirmed GIBD cases (− 15%). Although GCs were the most common initial treatment administered, use of TNFαi for BD and GIBD management increased by + 5.6% and + 8.1%, respectively. Severe AEs (mainly pneumonia and GI-associated AEs) were reported in 40% of patients receiving TNFαi; however, a high retention rate (of up to 80%) was observed 3 years after treatment initiation.

**Conclusion:**

The use of TNFαi for GIBD treatment has increased in Japan in recent years. Additional research is necessary to further evaluate TNFαi effectiveness in GIBD and other BD subtypes.

**Supplementary Information:**

The online version contains supplementary material available at 10.1007/s00535-024-02191-y.

## Introduction

Behçet’s disease (BD) is an autoinflammatory disease that affects multiple parts of the body [1–3]. Typical clinical features include oral and urogenital ulcers, as well as ocular and cutaneous lesions. Others include vascular, articular, neurological, and gastrointestinal (GI) manifestations [3].

Although the cause of BD is not currently known, both genetic and environmental factors play a role in disease pathogenesis. Genome-wide studies have identified multiple genes involved in the immune response as susceptibility factors, including HLA-B51, which is associated with the strongest predisposition. HLA-B51 expression varies depending on sex, with carriers being predominantly male [4, 5], and is associated with a higher incidence of ocular manifestations and lower prevalence of GI involvement, while its relation to genital ulceration remains unclear [4–8].

Environmental factors, such as geographical variation, have also been shown to impact BD frequency. BD exhibits a high prevalence in areas encompassing the Mediterranean Sea, the Middle East, and East Asia (China, Korea, and Japan), with the most significant occurrence found in Türkiye [9]. These regions also have the highest association between HLA-B51 and BD [8].

As there is currently no cure for BD, the primary goal of treatment is to alleviate symptoms and manage disease progression and severity [3, 10]. With anti-inflammatory agents, such as glucocorticoids (GCs) and/or immunosuppressants, these strategies focus on controlling inflammation, preventing irreversible tissue damage, and avoiding life-threatening complications [3, 11]. Targeting tumor necrosis factor α (TNF-α) with biological agents has made a substantial contribution to disease management, particularly in patients with severe organ involvement or resistance to conventional medications [12, 13].

Gastrointestinal Behçet’s disease (GIBD) is one of the most severe subtypes of BD and is associated with higher morbidity and mortality [3]. Data from different epidemiological studies suggest that between 15% and 50% of patients with BD in Japan have GI involvement [7, 14]. Patients suffering from GIBD present with lesions in the GI tract, most commonly located in the terminal ileum and ileocecal region. These lesions lead to abdominal pain, vomiting, weight loss, diarrhea, and bloody stool, with major complications that include intestinal bleeding and perforation [2, 14, 15]. TNF-α inhibitors (TNFαi) adalimumab and infliximab were approved for GIBD management in Japan in 2013 and 2015, respectively, and use for patients who fail to respond to conventional treatments has been incorporated into recent updates of the Japanese BD guidelines [12, 16–19].

The aim of this study was to analyze the prevalence and incidence of BD and GIBD and assess treatment patterns, especially the use of biologic agents (TNFαi), in these indications among patients in Japan with data recorded in the Japan Medical Data Center Claims Database (now JMDC Inc.).

## Methods

We conducted a retrospective descriptive observational study using the JMDC Claims Database (study period: 2017–2021). This repository includes data from multiple employee-based health insurance firms, covering civil servants, those employed in the private sector, and their respective families, and represents one of the major datasets for the working-age population in Japan [20]. Approximately 14,000,000 patients were included in this database as of 2022, accounting for about 10% of the Japanese population.

The database provides individual-level patient and clinical information, including demographics, date-stamped inpatient, and outpatient health insurance claims (using disease names for insurance purposes), diagnosis coded using the International Statistical Classification of Diseases and Related Health Problems, 10th revision [ICD-10], procedures, prescriptions, medical services, costs, and medical institutions, as well as health check-up records for some individuals [20].

No ethics approval from an institutional review board was required, as the study used pre-existing, anonymized data whose identifiable personal information could not be reconstructed. The study was, therefore, considered to be outside the scope of the Japanese Ethical Guidelines for Medical and Health Research Involving Human Subjects. In addition, no informed consent from the individuals included in this study was required, as all personal and site information were anonymized by JMDC Inc.

Patients were included in the study when they met the following criteria: recorded diagnosis of BD during the study period, as per the ICD-10 coding (M35.2). The cohort of patients who had at least one confirmed M35.2 diagnosis was considered for the prevalence and incidence analysis. Suspected cases were initially identified by symptom-based codes (e.g., codes for oral ulcers, ocular inflammation), but were not directly coded as M35.2 until further evaluation confirmed the diagnosis. Symptoms used to supplement the GIBD subtype were intestinal ulcer (ICD-10 code K633) and GI bleeding. BD subtype definition and drugs included in this study are described in Table 1.

**Table 1:** Definition of BD subtypes (a) and drugs included in the study (b)

(a) Definition of BD subtypes	
BD subtype	Disease code
Total BD	M35.2^a^
No specific BD subtype	
Articular feature	
Incomplete feature	8846052
Ocular feature	8845881
Oral feature	1361010
Genital feature	1361011
Specific BD subtype	
GI feature	8842203
Neurological feature	1361005
Vascular feature	1361009

(b) Drugs used		
Biologics	TNFαi	Adalimumab and infliximab
Non-biologics	GCs	Prednisolone and systemic GCs (H02A)
	CNi	Tacrolimus hydrate
	Immunomodulators	AZA, mercaptopurine hydrate, MTX and ciclosporin A
	Other	Apremilast, 5-ASA and colchicine

5-ASA, 5-aminosalicylic acid; AZA, azathioprine; BD, Behçet’s disease; CNi, calcineurin inhibitors; GCs, systemic glucocorticoids; GI, gastrointestinal; ICD-10, International Statistical Classification of Diseases and Related Health Problems, 10th revision; MTX, methotrexate; TNFαi, TNF-α inhibitors

^a^ICD-10 code M35.2 is used for BD diagnosis, while subtypes are defined via specified disease codes

Data analyses were performed with the intent-to-collect information on BD and GIBD prevalence and incidence as well as drug treatment patterns within the database, with special focus on TNFαi use and reported adverse events (AEs). Assessments were performed per fiscal year (FY), which comprised the period between April of the indicated year and March of the following year (e.g., FY2021 included data from April 2021 to March 2022). Some analyses were conducted for a single FY, while others compare over time dating back to 2017 or 2019, as indicated in each section and figure.

Prevalence and incidence were calculated as follows:

- Prevalence: simple annual aggregation by age/sex.
- Incidence: first M35.2 diagnosis in each patient was counted and patients who had any BD diagnosis within the first 6-month window in their medical record were excluded from the count, to ensure first diagnoses within the dataset.
- Scaling: patient counts for prevalence and incidence were scaled up to enable sizing of the Japanese BD patients and a scaling algorithm was applied to each sex and age segment to obtain estimates for 100% of the Japanese population.

## Results

### BD and GIBD demographics

#### BD subtypes, age, and sex distribution

**Fig. 1:**
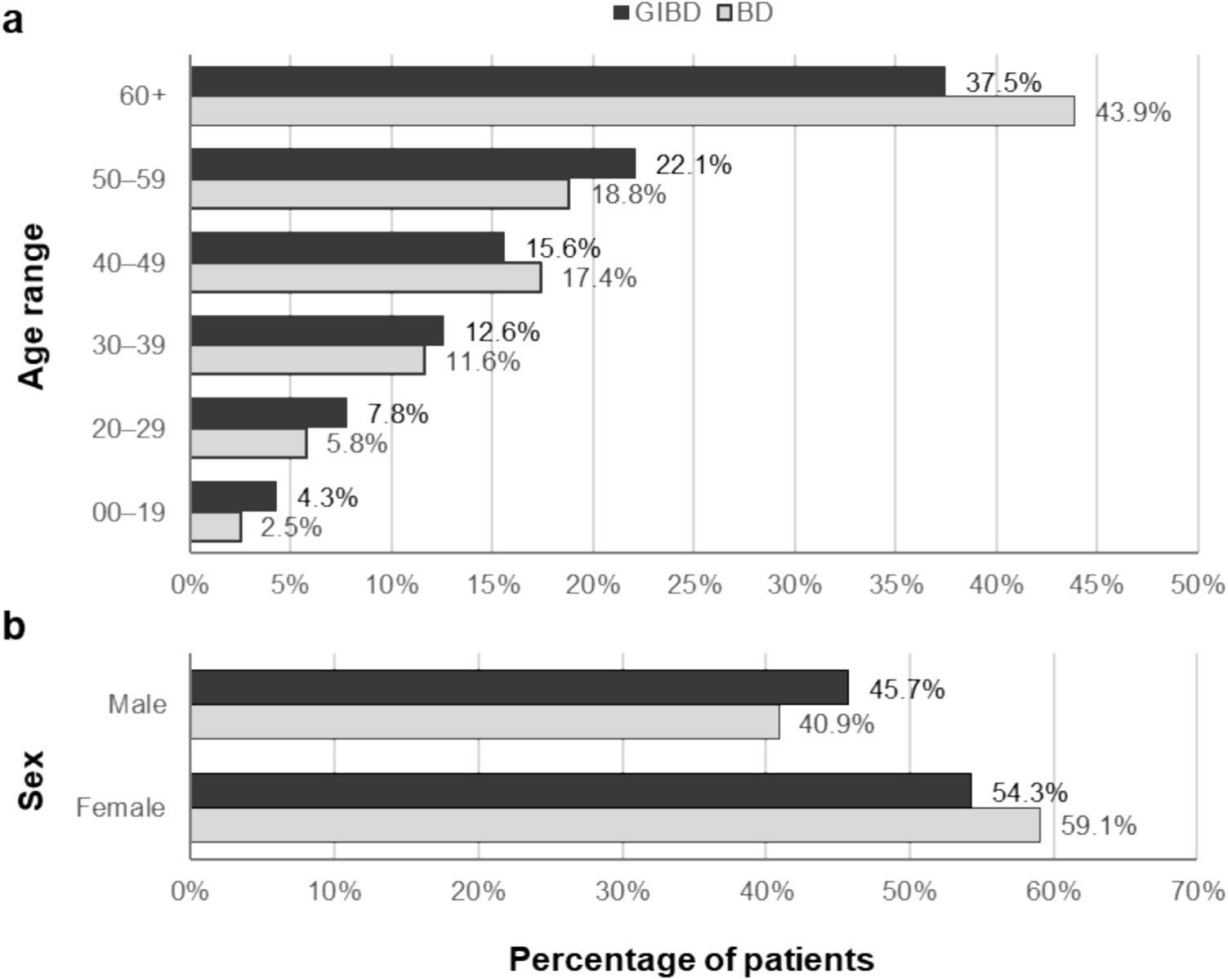
Demographics. BD and GIBD patient distribution according to age (**a**) and sex (**b**) in FY2021. Percentages may not add up to 100 due to rounding. Raw number is scaled to national level based on age and sex adjustment. BD, Behçet’s disease; FY, fiscal year; GI, gastrointestinal; GIBD, gastrointestinal Behçet’s disease

According to the JMDC dataset, 415 of 1993 patients diagnosed with BD during FY2021 had a specific BD subtype (GI, neurological, and vascular) (21%), with the most common being GIBD (14%) (Table 2). A high proportion of patients diagnosed with both BD and GIBD during FY2021 were ≥ 60 years of age (43.9% and 37.5%, respectively). The GIBD population was younger than the BD patient population (Fig. 1a), while there was a slightly higher proportion of female patients in both the BD and GIBD populations (59.1% and 54.3%, respectively) (Fig. 1b).

**Table 2:** Number of patients in the JMDC dataset diagnosed with a specific BD subtype

Number of patients with BD (%)^a^	
Total BD	1993 (100)
No specific BD subtype	1578 (79)
Articular feature	1244 (62)
Incomplete feature	200 (10)
Ocular feature	87 (4)
Oral feature	25 (1)
Genital feature	22 (1)
Specific BD subtype	415 (21)
GI feature	278 (14)
Neurological feature	101 (5)
Vascular feature	36 (2)

BD, Behçet’s disease; GI, gastrointestinal; JMDC, Japan Medical Data Center Claims

^a^Percentages may not add up to 100 due to rounding

#### BD and GIBD prevalence and incidence

**Fig. 2:**
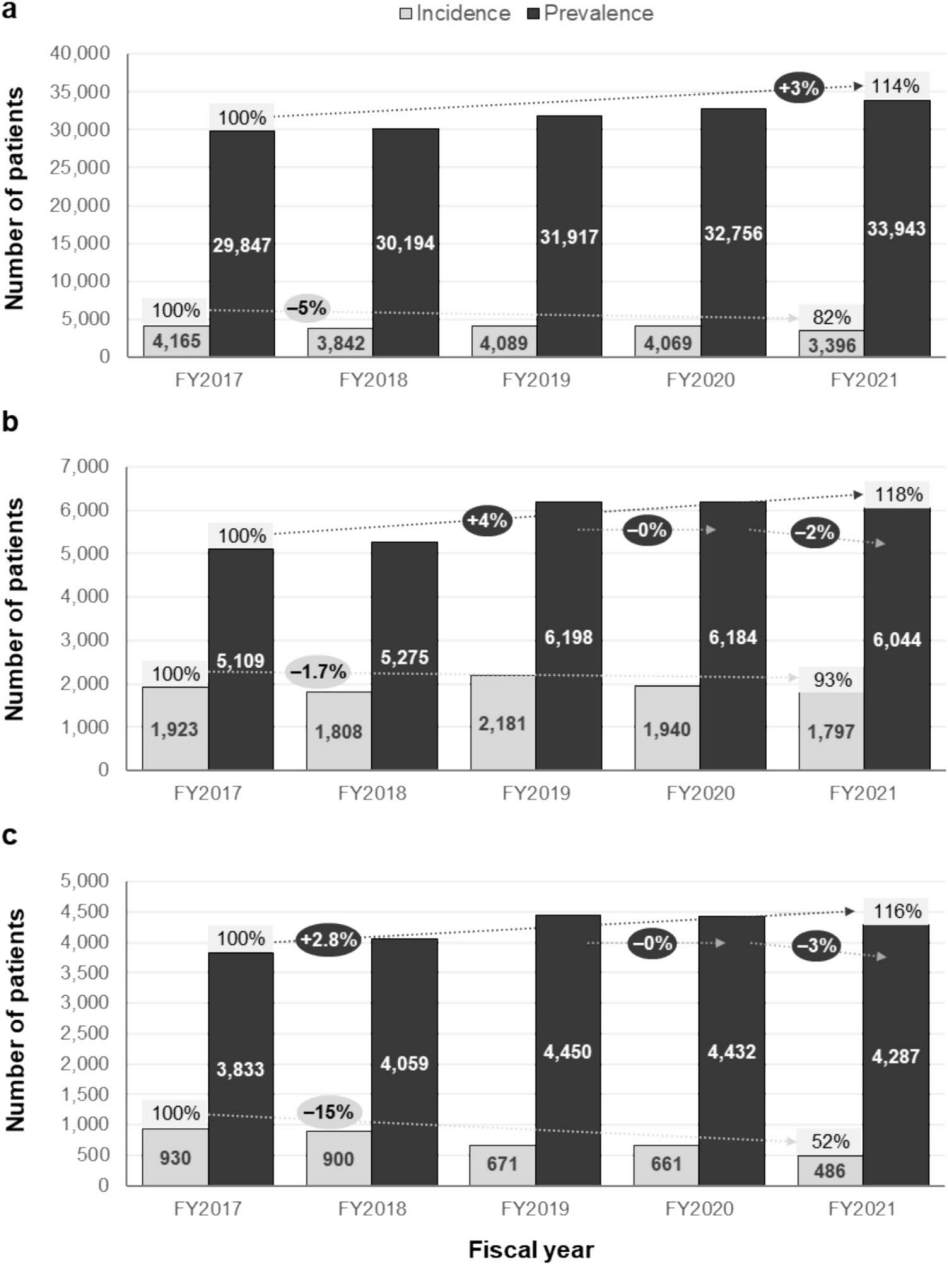
BD and GIBD prevalence and incidence. Prevalence and incidence of total BD (**a**), GIBD (including suspected cases (**b**) and GIBD (only confirmed cases) (**c**) between FY2107 and FY2021, as captured in the JMDC dataset. Raw number is scaled to national level based on age and sex adjustment. BD, Behçet’s disease; FY, fiscal year; GIBD, gastrointestinal Behçet’s disease; JMDC, Japan Medical Data Center Claims

During FY2021, there were a total of 33,943 patients with BD and 6044 patients with GIBD (including suspected cases) captured in the JMDC dataset, with over half of newly diagnosed BD patients (1797 out of 3396) having GIBD (Fig. 2). In general, BD prevalence in the database increased at an annual rate of + 3% between FY2017 and FY2021, while incidence decreased at an annual rate of − 5% (Fig. 2a). In contrast, although GIBD prevalence increased by + 4% between FY2017 and FY2021, it decreased by − 2% in the latest year, while incidence decreased at an annual rate of − 1.7% between FY2017 and FY2021 (Fig. 2b). Confirmed GIBD cases comprised 486 out of a total 1797 BD cases in FY2021 (Fig. 2c). 54% of patients with GIBD captured in the database between FY2017 and FY2021 had a confirmed diagnosis within 1 month after the first BD diagnosis, while 81% were confirmed within 3 months. In addition, the prevalence of confirmed GIBD cases decreased by − 3% from FY2020 to FY2021, while the incidence declined at a pronounced annual rate of − 15% between FY2017 and FY2021 (Fig. 2c).

### BD treatment patterns

#### Treatment in GIBD vs. non-GIBD

**Fig. 3:**
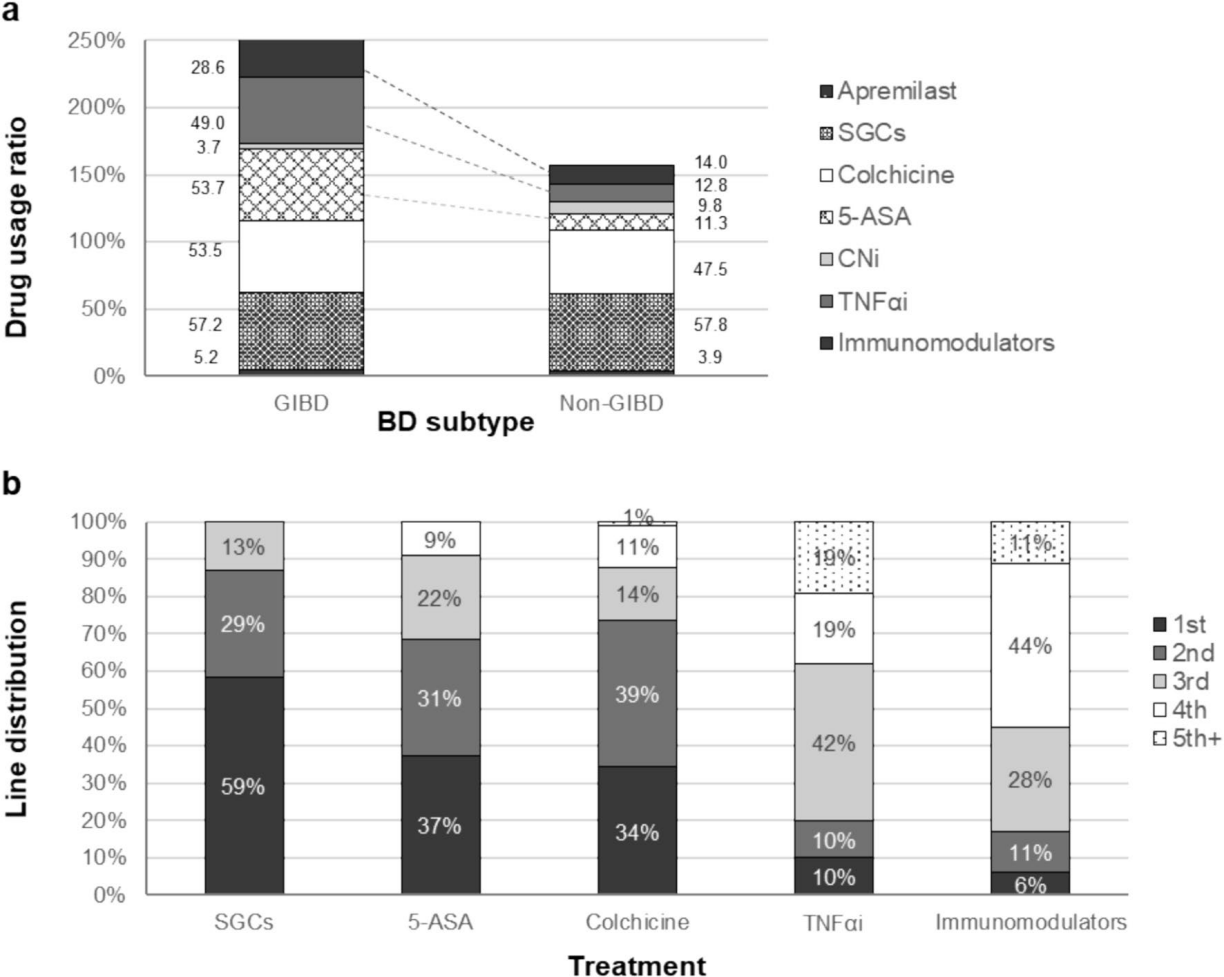
Drug usage. Comparison of drug usage ratio [including apremilast, SGCs (oral or intravenous), colchicine, 5-ASA, CNi, TNFαi, and other immunomodulators] between patients with GIBD and non-GIBD (**a**) and line distribution by treatment among GIBD patients (**b**), between FY2019 and FY2021. 5-ASA, 5-aminosalicylic acid; CNi, calcineurin inhibitors; GIBD, gastrointestinal Behçet’s disease; SGCs, systemic glucocorticoids; TNFαi, TNF-α inhibitors

Medical management was compared for patients with GIBD vs. non-GIBD (including other BD subtypes excluding GI involvement) over 3 years (FY2019–FY2021). Apremilast, systemic GCs (oral or intravenous), colchicine, 5-aminosalicylic acid (5-ASA), calcineurin inhibitors (CNi), TNFαi (adalimumab and infliximab), and other immunomodulators [including azathioprine (AZA), mercaptopurine hydrate, methotrexate (MTX), and ciclosporin A] were used to treat both subgroups, with immunomodulators, TNFαi, and 5-ASA use being substantially higher in patients with GIBD vs. those with non-GIBD (29% vs. 14%; 49% vs. 13%; 54% vs. 11%, respectively) (Fig. 3a). CNi and apremilast use was generally limited, suggesting a subordinate role in BD treatment in this population.

Approximately 40% of patients with GIBD were on line 3–5 of treatment, compared with only ~ 15% of patients with non-GIBD. While systemic GCs, 5-ASA, and colchicine were typically administered as earlier lines of treatment (first and second), TNFαi and other immunomodulators were commonly initiated as later lines (third and fourth, respectively). In particular, 42% of TNFαi treatment for patients with GIBD was initiated as third-line therapy (Fig. 3b).

#### Use of TNFαi biologics

**Fig. 4:**
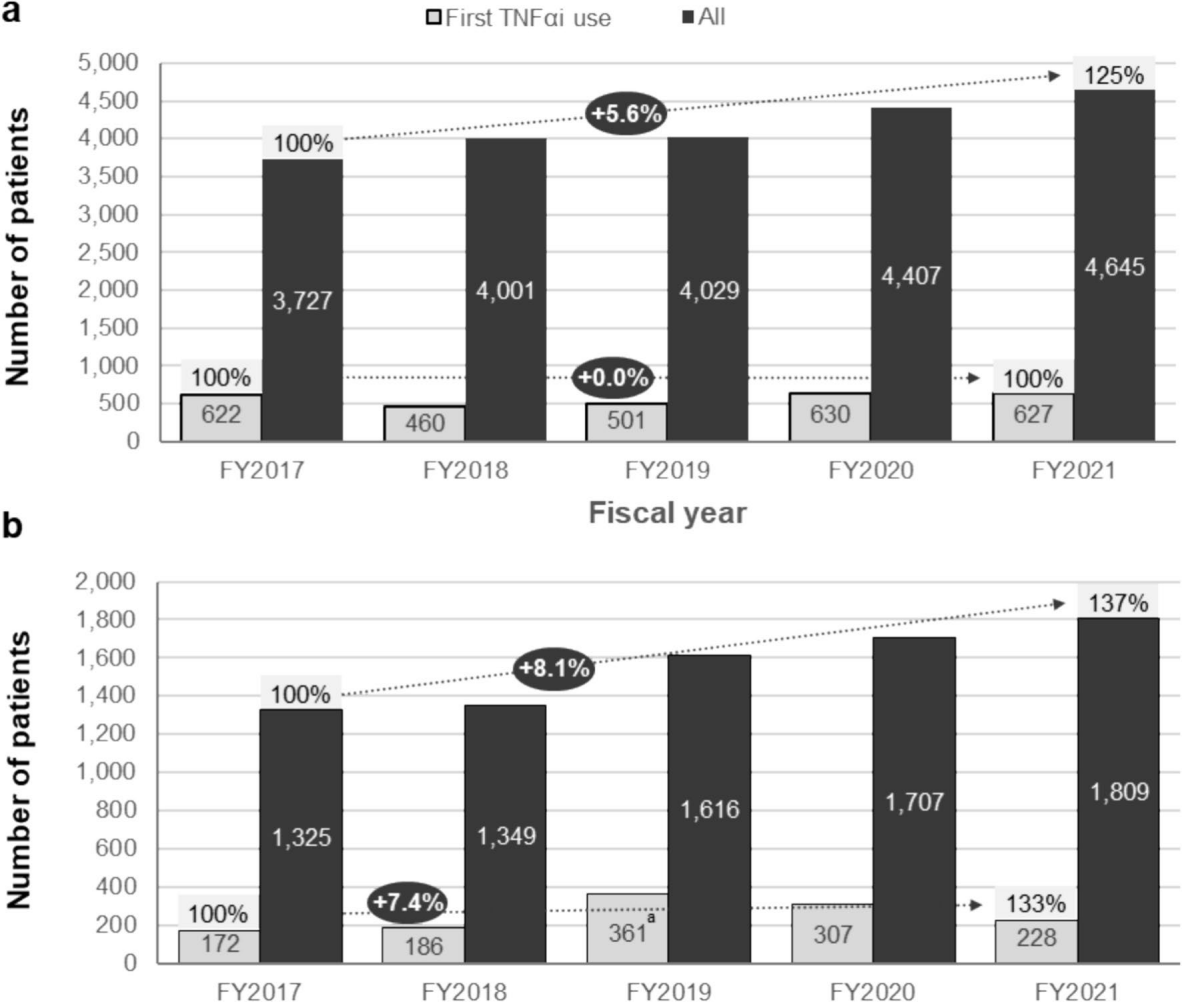
TNFαi biologics trends. TNFαi use for BD (**a**) and GIBD (confirmed cases only) (**b**) between FY2107 and FY2021, as captured in the JMDC dataset. ^a^N is small (10‒15), causing potential deviations. BD, Behçet’s disease; FY, fiscal year; GIBD, gastrointestinal Behçet’s disease; JMDC, Japan Medical Data Center Claims

According to the JMDC dataset, adalimumab use in Japanese patients with GIBD was slightly higher than infliximab use during FY2021 (55% vs. 45%). During FY2021, there were a total of 4645 patients with BD and 1809 patients with confirmed GIBD receiving TNFαi biologics, with 627 and 228 of those treated with a TNFαi for the first time, respectively. Use of TNFαi biologics in all patients with BD increased at an annual rate of + 5.6% between FY2017 and FY2021, while for patients receiving TNFαi treatment for the first time, it remained constant (Fig. 4a). TNFαi biologic use showed a + 8.1% and a + 7.4% increase between FY2017 and FY2021 for all patients with GIBD and those receiving a TNFαi for the first time, respectively (Fig. 4b). In parallel, GC use showed a − 6.3% and a − 8.2% decrease between FY2017 and FY2021 for these groups of patients.

**Fig. 5:**
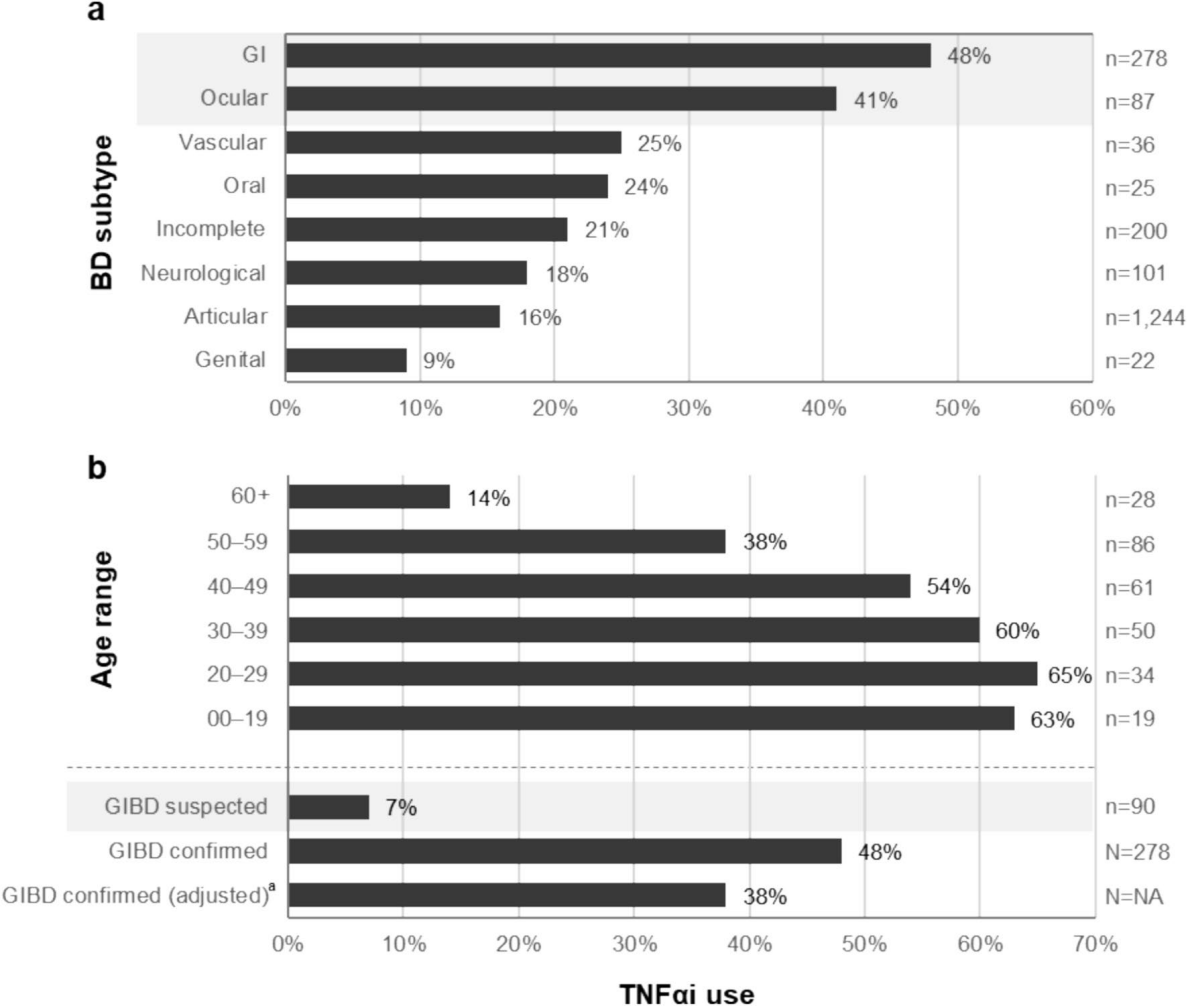
TNFαi biologics according to BD subtype and age. Use of TNFαi by BD subtype (**a**) and in patients with GIBD, depending on age and GIBD confirmed/suspected status (**b**), in FY2021. ^a^Age/gender adjusted to the national population in Japan. BD, Behçet’s disease; FY, fiscal year; GI, gastrointestinal; GIBD, gastrointestinal Behçet’s disease; TNFαi, TNF-α inhibitors

Use of biologic agents, adalimumab and infliximab, was highest among the GI (48%) and ocular (41%) BD subtypes, while for other forms of BD, such as vascular, oral, and neurological BD, treatment with TNFαi biologics was below 25% (Fig. 5a). In addition, treatment with biologics was lower in elderly patients with confirmed GIBD compared with younger patients. While over 50% of patients < 50 years of age received TNFαi biologics, only 38% and 14% of patients aged 50–59 and ≥ 60 years, respectively, received this treatment option (Fig. 5b). In general, TNFαi were prescribed to 48.2% of patients with confirmed GIBD, whereas biologic medication was rarely used to treat suspected cases (6.7%) (Fig. 5b). Moreover, use of biologics was particularly high in patients with confirmed GIBD and rheumatoid arthritis (RA) symptoms; 67% of patients experiencing these symptoms used TNFαi vs. 42% for those without RA symptoms.

#### TNFαi switching and co-medication

During their course of treatment, patients with GIBD may switch between TNFαi infliximab and adalimumab. In FY2021, 17.3% of patients were receiving treatment with a second TNFαi, with 5.2% of patients having switched from one TNFαi to the other during this period.

Patients may also receive a combination of treatments. During FY2021, only 18% of patients with GIBD received TNFαi treatment as monotherapy, while others received TNFαi in combination with GCs, 5-ASA, and/or immunomodulators (Fig. S1). The proportion of patients receiving TNFαi as a monotherapy was 16% when considering GCs as prednisolone only vs. 12% when expanding the definition to systemic GCs. Independent of the GC definition, most patients (81%) continued TNFαi treatment, with few initiating (5–6%) or discontinuing (13–14%) this treatment. In addition, over 90% of patients that discontinued TNFαi treatment continued taking other medications, with GCs (42%) and other immunomodulators (17%) as the most common options.

### TNFαi treatment assessment

#### TNFαi biologic treatment initiation and duration

A total of 22% of patients in the JMDC dataset initiated TNFαi treatment prior to a formal GIBD diagnosis, while 64% received biologics within 1 month of a confirmed diagnosis. Patients using TNFαi between FY2017 and FY2019 showed a high level of adherence to the treatment, with 83% and 78% of patients remaining on treatment at 1 and 3 years after TNFαi initiation, respectively. Only 5.1% and 4.3% of patients with BD and GIBD, respectively, discontinued TNFαi treatment per year between FY2019 and FY2021.

#### AEs associated with TNFαi treatment

Analyses of patients with GIBD receiving TNFαi biologics during FY2021 revealed that 40% experienced severe AEs. These included pneumonia (32%), GI AEs (15%), sepsis (5%), and intestinal perforation (2%). Several risk factors were associated with severe GI AEs, such as more advanced age, female sex, GIBD subtype, and earlier therapy lines or first-time exposure to biologic agents. In addition, 8.2% of patients who received TNFαi treatment underwent surgery during FY2021.

## Discussion

Our study presents an overview of BD and GIBD incidence over time and the current treatment landscape for the Japanese working-age population and their families captured in the JMDC Claims Database. Accordingly, a scaling algorithm was applied where appropriate to reflect the age and gender structure of the general Japanese population.

With over 30,000 patients, the JMDC database included a significantly higher number of patients with BD in FY2021 than the 20,035 people who received medical care for BD in 2014, according to the Ministry of Health, Labour, and Welfare [21]. These discrepancies can possibly be attributed to the inclusion of suspected or mild cases in the database. GIBD was the most common subtype (14%) detected among patients with BD during FY2021. Surprisingly, during this time, more than half of newly diagnosed patients presented with GI involvement (1797 out of 3396 patients). In Japan, a substantially high proportion of GIBD has been reported in the past. Although prevalence and incidence rates vary considerably between studies (12–50%) [14], caution should be exercised when comparing and interpreting the data in terms of timing (study conduct and observation period), source of data collection (e.g., registry, database vs. individual hospital/institution), patient characteristics (e.g., age, sex, disease onset), and geographical aspects. The data may also vary due to different and evolving diagnostic criteria applied, distinct clinical disciplines involved (e.g., gastroenterology, rheumatology), or misdiagnosis (i.e., lack of consideration of differential diagnoses such as Crohn’s disease or ulcerative colitis).

Slightly more female patients diagnosed with BD and GIBD were captured in the JMDC Claims Database, which was in accordance with other studies in Japanese and Korean populations [22, 23]. Further evidence from other Asian cohorts (from Türkiye and China) showed a more similar proportion of male and female patients with BD and GIBD, with a slightly higher proportion of males in the Chinese study [24, 25].

As described in previous studies, the frequency of patients with GI involvement reported in the JMDC Claims Database is higher than that of other regions from East Asia. Although countries such as Türkiye or Korea have a generally high BD incidence, only a small proportion of patients were reported to present GI lesions [2, 14]. In contrast, in other cohorts from Russia, the US, or the UK, which are known to have a lower BD incidence, a larger proportion of GIBD cases was observed [2, 14]. However, these data must be interpreted with caution, considering the different regional diagnostic criteria, and the possibility of misdiagnoses due to the lack of endoscopic assessments [14].

While GIBD might not be the most common BD subtype in other regions, an increasing trend in the presence of GI manifestations has been described in other Japanese and Korean studies. Research by Soejima and colleagues, who analyzed the clinical clusters contributing to the evolution of BD in Japan between 1991 and 2014, was based on data from a hospital-based regional BD registry as well as the Japanese national BD registry [7]. In this study, an increase in GI involvement in BD was observed, particularly after 2010 [7]. A similar trend regarding GI manifestations was also found in a Korean study, which analyzed a large hospital-based patient BD registry with data gathered between 1983 and 2012 [26]. Due to the similarities in the genetic backgrounds and previous diagnostic criteria in these countries [27], it is possible that at least some factors contributing to the prevalence of GI involvement in patients from East Asia are environmental. Interestingly, the adoption of a westernized lifestyle is believed to play a role in the recent expansion of other inflammatory bowel diseases in both Japan and Korea [28], which could also contribute to the predominance of GIBD.

In contrast, BD and overall GIBD incidence (including both confirmed and suspected GIBD cases) were shown to slightly decrease (annual rates of − 5% and − 1.7%, respectively) between FY2017 and FY2021, although this decline was more prominent for confirmed GIBD cases (− 15%). Changes in GIBD diagnostic parameters and therapeutic guidelines from the Japanese society for BD in 2020 [19] could be one of the key factors influencing this outcome, as the use of the proposed diagnostic algorithm is likely to help exclude false positives. These may also explain the discrepancies observed between the suspected and confirmed GIBD cases, with the latter being potentially underestimated. Moreover, improved hygiene in Japan, particularly during the SARS-CoV-2 pandemic, may have impacted GIBD occurrence. An alternative explanation is that GIBD was potentially underdiagnosed during the SARS-CoV-2 pandemic, due to restricted access to care. Interestingly, although both the SARS-CoV-2 virus and the COVID-19 vaccines have the potential to lead to systemic vasculitis, no events of BD post-infection or vaccination have been described [29]. The potential connection between SARS-CoV-2 (especially for the pre-Omicron viral variants) and the development of some autoimmune diseases, including ulcerative colitis, has been documented; whereas, other syndromes, such as Crohn’s disease, seem to be less likely to occur after a COVID-19 infection [30]. Other reports have shown minimal disease flares upon COVID-19 vaccination for some inflammatory and rheumatic diseases [31–33].

The extended use of TNFαi for BD treatment, as well as for other diseases (e.g., Crohn’s disease or ulcerative colitis), may have impacted the management of BD and its evolution. TNFαi administration increased both for BD and GIBD between FY2017 and FY2021 (annual rates of + 5.6% and + 8.1%, respectively). In particular, during FY2021, adalimumab use was slightly higher than infliximab, which could be due to infliximab being approved at a later timepoint than adalimumab for this indication [12, 16–19]. Compared with other subtypes, treatment with TNFαi biologics was highest for GIBD (48%), although it was observed that TNFαi were mainly prescribed to patients with confirmed GIBD, while they were less frequently used to treat suspected GIBD cases. A differential diagnosis of GIBD is complex due to similarities with other disorders, such as inflammatory bowel syndrome, Crohn’s disease, and ulcerative colitis [34], as well as simple ulcers in the GI tract, but a correct diagnosis is essential to ensure access to and maximum benefit from appropriate therapies [10].

Aside from the BD subtype, another aspect that was shown to influence the use of TNFαi was the medication previously used. TNFαi treatment for GIBD was often initiated in the third-line setting (in 42% of cases), which suggests that patients who receive TNFαi may be heavily pre-treated. In addition, only 12–18% of patients received TNFαi monotherapy, while the majority was administered TNFαi in combination with GCs, 5-ASA, and/or other immunomodulators. The information captured in the JMDC Claims database reflects the use of several immunomodulators, including AZA, mercaptopurine hydrate, MTX, and ciclosporin A. While thiopurines are widely employed to manage GIBD, with their use being recommended by the Japanese BD guidelines for patients who are dependent on GCs, or for those in whom GCs and/or TNFαi are ineffective, the efficacy of MTX remains unclear, and their use as monotherapy is not advised [19, 35]. Physicians’ specialization might influence immunomodulator administration, with MTX being more commonly prescribed among rheumatologists and AZA among GI specialists.

Given the secondary effects of long-term GC use and the promising efficacy results observed with TNFαi and other biologics, these agents could potentially become a GC-sparing therapy [12]. In accordance with this, a retrospective study based on patients with ulcerative colitis captured in the JMDC Claims Database showed a decrease in the use of GCs between 2006 and 2016, as treatment with biologics increased, although inappropriate use of GCs (such as long-term regimens) still persisted [36]. A recent study demonstrated that TNFαi monotherapy, or a TNFαi in combination with reduced GC doses, showed better potential for GIBD management in terms of GI ulcer healing and relative to GC monotherapy [37]. In this study, GC use also showed a decrease between FY2017 and FY2021 for patients with GIBD, which could be attributable to the introduction of biologic agents. The initial dosage remained stable over the study period (14 mg or 15 mg prednisolone, administered for a duration of 28–34 days ± 3 days). However, the lack of information related to the GC dosage over time limits the conclusions, notably about the tapering effect, and would need additional exploration [37]. Currently, although early introduction of TNFαi is encouraged in the Japanese BD guidelines [19], GIBD treatment in Japan still relies heavily on GCs, followed by immunomodulators and TNFαi.

Reimbursement access and the costs associated with TNFαi treatment may also affect the timing of biologic use in Japan, with TNFαi only available at earlier treatment stages for severe BD cases. Reimbursement benefits were also suggested to affect biologic use in Korea [38]. Moreover, the treatment patterns between 2011 and 2014 in Korea, as described by Han and colleagues, were similar to those in Japan, with GCs being the main therapeutic option (in 53.2% of patients), followed by immunomodulators (35.6%) and TNFαi biologics (4.7%) [38].

Treatment duration and AEs were also recorded in the JMDC Claims database. High adherence to TNFαi regimens was observed (around 80% after 3 years); however, a large proportion of patients experienced severe AEs (40%), especially related to infections. Previous systematic reviews have already identified increased vulnerability to infections as one of the main complications associated with TNFαi treatment [39–42]. Another complication, which is related to the severity of BD and is among the main causes of morbidity and mortality, is the appearance of intestinal perforations [14, 15]. In this study, intestinal perforations were observed in 2% of patients with GIBD receiving TNFαi biologics. This shows an important decline compared to the incidence reported in earlier studies, where these occurred in up to 13–25% of patients with GIBD [15, 43, 44]. The inclusion of BD treatments other than TNFαi, such as high-dose GCs, may have contributed to the risk of developing perforations in previous research [10]. In addition, differences in the patient population recruited across studies, and their predisposition to suffer intestinal perforations, could have also affected these results.

Surprisingly, in contrast to the high proportion of patients experiencing severe AEs, only 4.3% of patients with GIBD discontinued TNFαi treatment per year. The high retention rate suggests that, aside from a high efficacy, AEs associated with TNFαi use may generally be manageable [41, 42]. Nonetheless, further analyses comprising not only the working-age population and their families, but the general population, are needed to better understand the long-term AE profile of TNFαi and other biologic agents.

It is important to address the unresolved unmet need for the small group of patients that discontinue TNFαi treatment, but who could potentially benefit from other biologic agents or alternative treatments. Previous research has identified several clinical variables as predictors for GIBD prognosis. Variables such as younger age at diagnosis, history of surgery, and lack of initial response to medication, among others, have been linked to a poor prognosis [45]. Although these factors need to be further studied to fully understand their predictive power, they may be helpful tools for a more personalized and effective care, which could especially help subgroups of patients with these unmet needs. A more complete understanding of the genetic predisposition underlying GIBD, including HLA-B51 and other genetic factors, as well as investigation of emerging treatments for GIBD management, which may involve different targets aside from TNF-α, will also contribute to achieving these goals.

Some limitations are to be considered when interpreting the JMDC data, as only patients who visit hospitals or clinics and are covered by company health insurance are captured in the dataset. Since Japanese employees tend to remain insured with the same health insurance company for several years, which assigns them a personal ID, they can be followed over various time periods, even if they are treated by different medical facilities [46, 47].

Age and sex are included in the database, but it only comprises the working-age population and their families, i.e., the elderly population (60–75 years of age) is drastically underrepresented (≤ 3% vs. ~ 9–10% for younger age groups), which is important given that the overall Japanese population is aging. Accordingly, no comparative analyses, e.g., on drug use in elderly versus younger patients with BD, can be carried out with the necessary robustness based on the JMDC data, even if these are of scientific interest. Furthermore, direct comparisons with other studies and more representative populations may need to be interpreted with caution.

Another known limitation of the JMDC is the lack of information on disease severity, extension of inflammation and treatment outcome [46]. Accordingly, this study was not designed to analyze corresponding time trends related to any of these.

Furthermore, many AEs may not be captured in the database, and may, therefore, be underrepresented. In addition, patients with multiple comorbidities, including disorders that may also respond to TNFαi treatment, have the potential to be classified under another diagnosis and, therefore, not captured in this analysis.

Surgery rates have not been assessed in this manuscript due to limited data on whether patients have been followed and recorded from their first surgery, and whether they had subsequent surgeries.

Furthermore, it should be noted that the JMDC database collects data from Diagnosis Procedure Combination (DPC) and non-DPC hospitals nationwide, and the given dataset does not allow for sufficiently accurate analyses by geographical regions and prefectures [20].

In conclusion, while BD and GIBD prevalence slightly increased in Japan between 2017 and 2021, incidence declined, which was particularly notable for confirmed GIBD cases. In parallel, the use of TNFαi biologics for BD and GIBD management increased over the same period in Japan. This change in BD treatment patterns, along with the Japanese diagnostic criteria adjustment in 2020, may have impacted the observed decrease in GIBD incidence. Further studies are needed to better understand the effectiveness of TNFαi biologics to treat GIBD as well as other BD subtypes, and to help achieve a more personalized treatment approach. These strategies may improve the availability of TNFαi biologics in Japan, potentially replacing the long-term use of GCs, which are often still administered as the initial therapeutic option for BD management.

## Supplementary Information

Below is the link to the electronic supplementary material.

Supplementary file1 (DOCX 53 KB)

## References

[1] Adil A, Goyal A, Quint JM. Behcet disease. In: StatPearls. Treasure Island: StatPearls Publishing LLC; 2023. 29262080

[2] Nguyen A, Upadhyay S, Javaid MA, et al. Behcet’s disease: an in-depth review about pathogenesis, gastrointestinal manifestations, and management. Inflamm Intest Dis. 2021;6:175–85. 35083283 10.1159/000520696 PMC8740277

[3] Zeidan MJ, Saadoun D, Garrido M, et al. Behçet’s disease physiopathology: a contemporary review. Auto Immun Highlights. 2016;7:4. 26868128 10.1007/s13317-016-0074-1 PMC4751097

[4] de Menthon M, Lavalley MP, Maldini C, et al. HLA-B51/B5 and the risk of Behçet’s disease: a systematic review and meta-analysis of case-control genetic association studies. Arthritis Rheum. 2009;61:1287–96. 19790126 10.1002/art.24642 PMC3867978

[5] Maldini C, Lavalley MP, Cheminant M, et al. Relationships of HLA-B51 or B5 genotype with Behcet’s disease clinical characteristics: systematic review and meta-analyses of observational studies. Rheumatology (Oxford). 2012;51:887–900. 22240504 10.1093/rheumatology/ker428

[6] Mizuki Y, Horita N, Horie Y, et al. The influence of HLA-B51 on clinical manifestations among Japanese patients with Behçet’s disease: a nationwide survey. Mod Rheumatol. 2020;30:708–14. 31386589 10.1080/14397595.2019.1649103

[7] Soejima Y, Kirino Y, Takeno M, et al. Changes in the proportion of clinical clusters contribute to the phenotypic evolution of Behçet’s disease in Japan. Arthritis Res Ther. 2021;23:49. 33522943 10.1186/s13075-020-02406-6 PMC7851921

[8] Takeno M. The association of Behçet’s syndrome with HLA-B51 as understood in 2021. Curr Opin Rheumatol. 2022;34:4–9. 34690278 10.1097/BOR.0000000000000846 PMC8635258

[9] Mahmoudi M, Aslani S, Meguro A, et al. A comprehensive overview on the genetics of Behçet’s disease. Int Rev Immunol. 2022;41:84–106. 33258398 10.1080/08830185.2020.1851372

[10] Hatemi G, Christensen R, Bang D, et al. 2018 update of the EULAR recommendations for the management of Behçet’s syndrome. Ann Rheum Dis. 2018;77:808–18. 29625968 10.1136/annrheumdis-2018-213225

[11] Alpsoy E, Leccese P, Emmi G, et al. Treatment of Behçet’s disease: an algorithmic multidisciplinary approach. Front Med (Lausanne). 2021;8: 624795. 33996847 10.3389/fmed.2021.624795 PMC8115406

[12] Bozca BC, Alpsoy E. Experimental therapeutic solutions for Behcet’s disease. J Exp Pharmacol. 2021;13:127–45. 33603502 10.2147/JEP.S265645 PMC7886245

[13] Kinoshita H, Kunisaki R, Yamamoto H, et al. Efficacy of infliximab in patients with intestinal Behçet’s disease refractory to conventional medication. Intern Med. 2013;52:1855–62. 23994973 10.2169/internalmedicine.52.0589

[14] Cheon JH, Hatemi I, Çelik AF, et al. Behçet syndrome: gastrointestinal involvement. In: Yazici Y, Hatemi G, Seyahi E, et al., editors. Behçet syndrome. Cham: Springer International Publishing; 2020, pp. 117–41.

[15] Skef W, Hamilton MJ, Arayssi T. Gastrointestinal Behçet’s disease: a review. World J Gastroenterol. 2015;21:3801–12. 25852265 10.3748/wjg.v21.i13.3801 PMC4385527

[16] Japanese Pharmaceuticals and Medical Devices Agency. https://www.pmda.go.jp/english/. Accessed 26 Mar 2024.

[17] Hibi T, Hirohata S, Kikuchi H, et al. Infliximab therapy for intestinal, neurological, and vascular involvement in Behcet disease: efficacy, safety, and pharmacokinetics in a multicenter, prospective, open-label, single-arm phase 3 study. Medicine (Baltimore). 2016;95: e3863. 27310969 10.1097/MD.0000000000003863 PMC4998455

[18] Ueda A, Takeno M, Ishigatsubo Y. Adalimumab in the management of Behçet’s disease. Ther Clin Risk Manag. 2015;11:611–9. 25926738 10.2147/TCRM.S56163 PMC4403514

[19] Watanabe K, Tanida S, Inoue N, et al. Evidence-based diagnosis and clinical practice guidelines for intestinal Behçet’s disease 2020 edited by Intractable Diseases, the Health and Labour Sciences Research Grants. J Gastroenterol. 2020;55:679–700. 32377946 10.1007/s00535-020-01690-y PMC7297851

[20] JMDC Claims Database: JMDC. https://www.jmdc.co.jp/en/jmdc-claims-database/. Accessed 27 Nov 2023.

[21] Kirino Y, Nakajima H. Clinical and genetic aspects of Behçet’s disease in Japan. Intern Med. 2019;58:1199–207. 30626832 10.2169/internalmedicine.2035-18 PMC6543215

[22] Ishido T, Horita N, Takeuchi M, et al. Clinical manifestations of Behçet’s disease depending on sex and age: results from Japanese nationwide registration. Rheumatology (Oxford). 2017;56:1918–27. 28968732 10.1093/rheumatology/kex285

[23] Jung YS, Yoon JY, Hong SP, et al. Influence of age at diagnosis and sex on clinical course and long-term prognosis of intestinal Behcet’s disease. Inflamm Bowel Dis. 2012;18:1064–71. 21793128 10.1002/ibd.21833

[24] Gürbüz C, Yalçın Kehribar D, Özgen M. Clinical manifestations of Behçet’s syndrome: a single-center cohort of 777 patients. Eur J Rheumatol. 2021;8:211–6. 35110181 10.5152/eurjrheum.2020.21199 PMC10176235

[25] Zou J, Luo JF, Shen Y, et al. Cluster analysis of phenotypes of patients with Behçet’s syndrome: a large cohort study from a referral center in China. Arthritis Res Ther. 2021;23:45. 33514418 10.1186/s13075-021-02429-7 PMC7847001

[26] Kim DY, Choi MJ, Cho S, et al. Changing clinical expression of Behçet disease in Korea during three decades (1983–2012): Chronological analysis of 3674 hospital-based patients. Br J Dermatol. 2014;170:458–61. 24117362 10.1111/bjd.12661

[27] Mizushima Y. Revised diagnostic criteria for Behçet’s disease in 1987. Ryumachi [Rheumatism]. 1988;28:66–70. 3388149

[28] Ng WK, Wong SH, Ng SC. Changing epidemiological trends of inflammatory bowel disease in Asia. Intest Res. 2016;14:111–9. 27175111 10.5217/ir.2016.14.2.111 PMC4863044

[29] Mv P, Auanassova A, Yessirkepov M, et al. New-onset systemic vasculitis following SARS-CoV-2 infection and vaccination: the trigger, phenotype, and outcome. Clin Rheumatol. 2023;42:2761–75. 37422611 10.1007/s10067-023-06694-6

[30] Hileman CO, Malakooti SK, Patil N, et al. New-onset autoimmune disease after COVID-19. Front Immunol. 2024;15:1337406. 38390319 10.3389/fimmu.2024.1337406 PMC10883027

[31] Barbhaiya M, Levine JM, Bykerk VP, et al. Systemic rheumatic disease flares after SARS-CoV-2 vaccination among rheumatology outpatients in New York City. Ann Rheum Dis. 2021;80:1352–4. 34158370 10.1136/annrheumdis-2021-220732

[32] Barbhaiya M, Levine JM, Siegel CH, et al. Adverse events and disease flares after SARS-CoV-2 vaccination in patients with systemic lupus erythematosus. Clin Rheumatol. 2022;41:1619–22. 34716843 10.1007/s10067-021-05963-6 PMC8556788

[33] Spinelli FR, Favalli EG, Garufi C, et al. Low frequency of disease flare in patients with rheumatic musculoskeletal diseases who received SARS-CoV-2 mRNA vaccine. Arthritis Res Ther. 2022;24:21. 35016701 10.1186/s13075-021-02674-w PMC8748531

[34] He K, Yan X, Wu D. Intestinal Behcet’s disease: a review of the immune mechanism and present and potential biological agents. Int J Mol Sci. 2023;24:8176. 37175882 10.3390/ijms24098176 PMC10179024

[35] Hisamatsu T, Hayashida M. Treatment and outcomes: medical and surgical treatment for intestinal Behçet’s disease. Intest Res. 2017;15:318–27. 28670228 10.5217/ir.2017.15.3.318 PMC5478756

[36] Matsuoka K, Igarashi A, Sato N, et al. Trends in corticosteroid prescriptions for ulcerative colitis and factors associated with long-term corticosteroid use: analysis using Japanese claims data from 2006 to 2016. J Crohns Colitis. 2021;15:358–66. 32845311 10.1093/ecco-jcc/jjaa172 PMC7944504

[37] Miyagawa I, Nakano K, Iwata S, et al. Comparative study of corticosteroid monotherapy, and TNF inhibitors with or without corticosteroid in patients with refractory entero-Behcet’s disease. Arthritis Res Ther. 2019;21:151. 31228955 10.1186/s13075-019-1933-8 PMC6589167

[38] Han M, Jung YS, Kim WH, et al. Incidence and clinical outcomes of intestinal Behçet’s disease in Korea, 2011–2014: a nationwide population-based study. J Gastroenterol. 2017;52:920–8. 28028610 10.1007/s00535-016-1300-3

[39] Cheon JH. Understanding the complications of anti-tumor necrosis factor therapy in East Asian patients with inflammatory bowel disease. J Gastroenterol Hepatol. 2017;32:769–77. 27723166 10.1111/jgh.13612

[40] Park J, Cheon JH. Anti-tumor necrosis factor therapy in intestinal Behçet’s disease. Gut Liver. 2018;12:623–32. 29788675 10.5009/gnl17462 PMC6254627

[41] Zhang M, Liu J, Liu T, et al. The efficacy and safety of anti-tumor necrosis factor agents in the treatment of intestinal Behcet’s disease, a systematic review and meta-analysis. J Gastroenterol Hepatol. 2022;37:608–19. 34894004 10.1111/jgh.15754

[42] Zhang Q, Ma C, Dong R, et al. Efficacy and safety of anti-tumor necrosis factor-alpha agents for patients with intestinal Behcet’s disease: a systematic review and meta-analysis. Yonsei Med J. 2022;63:148–57. 35083900 10.3349/ymj.2022.63.2.148 PMC8819411

[43] Chou SJ, Chen VT, Jan HC, et al. Intestinal perforations in Behçet’s disease. J Gastrointest Surg. 2007;11:508–14. 17436137 10.1007/s11605-006-0031-9 PMC1852375

[44] Moon CM, Cheon JH, Shin JK, et al. Prediction of free bowel perforation in patients with intestinal Behçet’s disease using clinical and colonoscopic findings. Dig Dis Sci. 2010;55:2904–11. 20094787 10.1007/s10620-009-1095-7

[45] Park JJ, Kim WH, Cheon JH. Outcome predictors for intestinal Behçet’s disease. Yonsei Med J. 2013;54:1084–90. 23918555 10.3349/ymj.2013.54.5.1084 PMC3743188

[46] Nagai K, Tanaka T, Kodaira N, et al. Data resource profile: JMDC claims database sourced from health insurance societies. J Gen Fam Med. 2021;22:118–27. 33977008 10.1002/jgf2.422 PMC8090843

[47] Laurent T, Simeone J, Kuwatsuru R, et al. Context and considerations for use of two Japanese real-world databases in Japan: Medical Data Vision and Japanese Medical Data Center. Drugs Real World Outcomes. 2022;9:175–87. 35304702 10.1007/s40801-022-00296-5 PMC8932467

