# Supplementary material for: Behçet’s disease: incidence, prevalence, and real-word data on the use of biologic agents in Japan
Source: J Gastroenterol. 2024 Dec 6;60(3):294–305. doi: 10.1007/s00535-024-02191-y (PMC11880103; doi:10.1007/s00535-024-02191-y)
Supplement: Supplementary file 1 — Supplementary file1 (DOCX 53 KB) [file 535_2024_2191_MOESM1_ESM.docx]

# Supplementary Information for:

Title: Behçet's Disease: Incidence, Prevalence and Real-Word Data on the Use of Biologic Agents in Japan

Journal: Journal of Gastroenterology

Authors: Tadakazu Hisamatsu^1^, Makoto Naganuma^2^, Philippe Pinton^3^ and Mitsuhiro Takeno^4^

Affiliations: ^1^Department of Gastroenterology and Hepatology, Kyorin University School of Medicine, Tokyo, Japan; [thisamatsu@ks.kyorin-u.ac.jp](mailto:thisamatsu@ks.kyorin-u.ac.jp); ^2^Third Department of Internal Medicine, Division of Gastroenterology and Hepatology, Kansai Medical University, Osaka, Japan; [naganuma@hirakata.kmu.ac.jp](mailto:naganuma@hirakata.kmu.ac.jp); ^3^Clinical and Translational Sciences, Ferring Pharmaceuticals, 2770 Kastrup, Denmark; [philippe.pinton@ferring.com](mailto:philippe.pinton@ferring.com)

^4^Department of Allergy and Rheumatology, Nippon Medical School Musashi Kosugi Hospital, Kanagawa, Japan; [m-takeno@nms.ac.jp](mailto:m-takeno@nms.ac.jp);

E-mail address of the corresponding author: [m-takeno@nms.ac.jp](mailto:m-takeno@nms.ac.jp).

**Fig. S1**


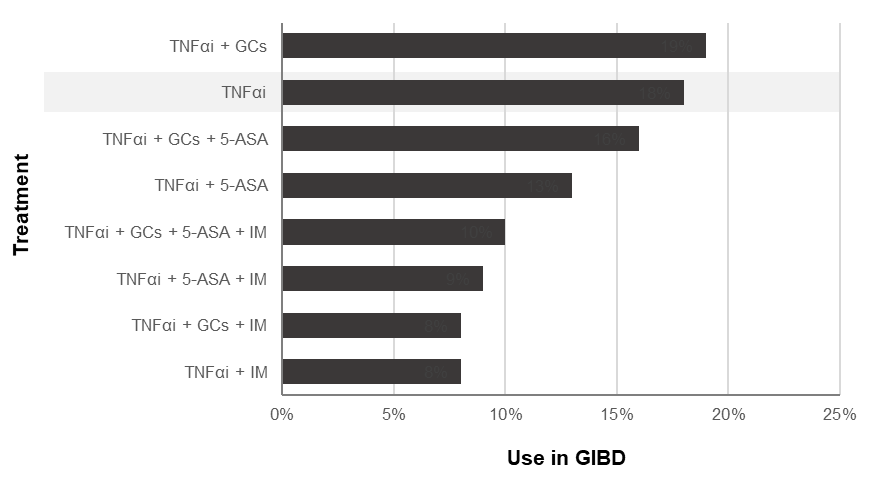


**Co-medication status.** Use of TNFαi as monotherapy and in combination with other treatment options (GCs, 5-ASA and/or IM) for patients with GIBD in FY2021.

5-ASA, 5-aminosalicylic acid; FY, fiscal year; GCs, glucocorticoids; GIBD, gastrointestinal Behçet’s disease; IM, immunomodulators; TNFαi, TNF-α inhibitors
